# Increased expression of nucleophosmin/B23 in hepatocellular carcinoma and correlation with clinicopathological parameters

**DOI:** 10.1038/sj.bjc.6603574

**Published:** 2007-01-23

**Authors:** J-P Yun, J Miao, G G Chen, Q-H Tian, C-Q Zhang, J Xiang, J Fu, P B S Lai

**Affiliations:** 1 State Key Laboratory of Oncology in Southern China, Cancer Center, Sun Yat-Sen University, Guangzhou, China; 2 Department of Pathology, Cancer Center, Sun Yat-Sen University, Guangzhou, China; 3 Department of Surgery, The Chinese University of Hong Kong, SAR Hong Kong, China

**Keywords:** nucleophosmin, B23, hepatocellular carcinoma, PCNA, cell proliferation, malignancy

## Abstract

Nucleophosmin (NPM, B23, numatrin, NO38) is an abundant nucleolar phosphoprotein involved in multiple cellular functions. Previous evidence indicates that high-level expression of NPM causes uncontrolled cell growth and suggests that NPM may have oncogenic potential. In this study, we examined NPM expression in 103 paired cases of hepatocellular carcinoma (HCC), 12 cases of hepatic focal nodular hyperplasia, 17 cases of liver tissue adjacent to a hepatic haemangioma, and series of array tissues from normal human organs and malignancies using a monoclonal antibody against NPM and reverse transcription–PCR techniques, Western blot analysis, immunohistochemistry, and immunocytofluorescence. Our data indicated that NPM expression was significantly higher in HCC than in the non-malignant hepatocytes (*P*<0.001). Nucleophosmin was weakly expressed in hepatocytes from a 5-month-old embryo and in stationary hepatocytes of healthy adults. Moreover, enhanced expression of NPM in HCC correlated with the level of PCNA (*R*^2^=0.5639) and with the clinical prognostic parameters such as serum alpha fetal protein level, tumour pathological grading, and liver cirrhosis (*P*<0.05). Our results suggest that NPM may play an important role in the progression of tumorigenesis and that NPM may serve as a potential marker for HCC.

Hepatocellular carcinoma (HCC) is one of the most prevalent malignancies worldwide, with an annual incidence of 250 000 diagnoses. Although the process of hepatocarcinogenesis has not yet been fully elucidated, it is considered a multifactorial and multistep process that involves the activation of oncogenes or the inactivation of tumour suppressor genes during the different stages of HCC progression. Several studies have recently shown that activation of oncogenes was involved in hepatocarcinogenesis, genes such as cyclin D1 (Deane *et al*, 2001), the transcription factor Yin Yang 1 (YY1) (Gordon *et al*, 2006), and the hedgehog pathway (Sicklick *et al*, 2006). Overexpression of cyclin D1 has been shown to be sufficient for the initiation of hepatocellular carcinogenesis in a transgenic mouse model (Deane *et al*, 2001). The transcription factor YY1 is considered to possess the potential to act as an initiator of tumorigenesis and may serve as both a diagnostic and prognostic tumour marker (Gordon *et al*, 2006). Overexpression and tumorigenic activation of the Smoothened (SMO) proto-oncogene mediates c-myc overexpression, suggesting that SMO may also be a prognostic factor in HCC tumorigenesis (Sicklick *et al*, 2006).

Nucleophosmin (NPM) is a major nucleolar phosphoprotein implicated in multiple cellular functions, including ribosomal protein assembly and transport (Verheggen *et al*, 2000; Huang *et al*, 2005), centrosome duplication (Okuda *et al*, 2000; Okuda, 2002; Grisendi *et al*, 2005), molecular chaperone activity in preventing protein aggregation (Hingorani *et al*, 2000; Szebeni *et al*, 2003), and regulating the activity of the tumour suppressors p53 (Colombo *et al*, 2002; Li *et al*, 2004; Maiguel *et al*, 2004) and p14^ARF^ (Itahana *et al*, 2003; Bertwistle *et al*, 2004; Brady *et al*, 2004). Earlier studies have shown that the level of NPM is markedly and promptly increased in association with cell commitment to mitogenesis (Feuerstein and Mond, 1987; Feuerstein *et al*, 1988). It has also been shown that NPM is a direct target of the Myc transcription factor during ribosomal biogenesis (Zeller *et al*, 2001) and cyclin E/CDK2 kinase activity during centrosome duplication (Okuda *et al*, 2000). Nucleophosmin physically interacts with several other cellular factors such as the retinoblastoma susceptibility gene product pRB (Takemura *et al*, 1994, 1999), the cell cycle G2–M checkpoint factor Gadd45a (Gao *et al*, 2005), and the tumour suppressor p14^ARF^ (Itahana *et al*, 2003). Mounting evidence has linked excessive NPM to cellular transformation and ontogenesis (Pulford *et al*, 2001). Nucleophosmin overexpression is frequently found in human cancers, including cancers of the stomach (Tanaka *et al*, 1992), colon (Nozawa *et al*, 1996), bladder (Tsui *et al*, 2004), prostate (Subong *et al*, 1999), thyroid (Onda *et al*, 2004), ovary (Zhang, 2004), and myeloid and lymphoid cells (Schnittger *et al*, 2005). It has been shown that overexpression of NPM in bladder cancer is independently associated with cancer recurrence and progression to more advanced stages, suggesting that NPM overexpression is an important prognostic indicator for bladder cancer recurrence (Tsui *et al*, 2004). Furthermore, ectopic overexpression of NPM in NIH3T3 cells results in malignant transformation (Kondo *et al*, 1997). These findings further suggest that NPM is involved in the regulation of cellular growth in various normal and neoplastic cells and may be used as a clinical indicator for cancer patients.

The present study is aimed at investigating the potential roles of NPM in HCC using a systematic analysis on the levels of NPM in a large number of human HCC and non-malignant liver lesions, in normal human adult and embryo tissues, and to address the correlation between NPM expression and the clinical parameters.

## MATERIALS AND METHODS

### Nucleophosmin recombinant protein and monoclonal antibody

Human NPM was PCR amplified from cDNA (catalogue no. MHS1011-58590, clone ID: 2821577, OpenBioSystem, Huntsville, AL, USA) using specific primers according to NPM1 gene source sequence (5′-cgcggatccgaagattcgatggacatggac and 3′-cccaagcttaa gagacttcctccactgcca). The gene was then cloned into the Pqe30 vector (catalogue no. 33203, Qiagen, Valencia, CA, USA) and expressed in an *Escherichia coli* expression system to obtain recombinant NPM protein. The purified recombinant protein was used to generate a monoclonal antibody using routine hybridoma cell techniques. The titre and specificity of the antibodies were analysed and characterised by Western blot analysis and immunocytofluorescene. A PCNA monoclonal antibody was purchased (Zymed, South San Francisco, CA, USA).

### Clinical samples and tissue arrays

Samples from 132 cases with liver disease and their clinical material were collected from the files of the Cancer Center of Sun Yat-Sen University, Guangzhou, China. These cases included 103 cases of HCC, 12 cases of hepatic focal nodular hyperplasia (FNH), and 17 cases of hepatic haemangiomas. All of the tissue blocks were sectioned for immunohistochemistry of NPM and PCNA. Ten paired cases of HCC tissue along with the adjacent hepatic tissue from the tissue bank department of this cancer centre were collected for reverse transcription–PCR (RT–PCR) and Western blot analyses. Another tissue array with sections from multiple organs, including normal and diseased tissues for immunohistochemistry, was purchased from Cybrdi Biotech Co (catalogue no. CC00-11-002, CC00-11-003, CC00-01-004, EC01-01-006, NC03-01-001, Cybridi, Xian City, China). This array included normal adult tissues; normal embryo tissues (5 months); diseased tissues, including malignancies from liver, brain, lung, kidney, stomach, colon, breast, cervix, prostate, and skin; as well as cells from different origins such as epithelia, non-epithelia, and lymphocytes. All of the human specimens in the study were approved by the Independent Ethics Committee of the Cancer Center of Sun Yat-Sen University.

### Cell culture, immunocytofluorescence, and Western blot analysis

Six hepatoma cell lines including Hep G2, Huh-7, PLC/PRF/5, SK-Hep-1, Chang, and Hep-3B were cultured as reported previously (Yun *et al*, 2003; Miao *et al*, 2006). Briefly, for immunocytofluorescence, the cultured cells were fixed for 10 min and rinsed with PBS. The cells were then incubated with the primary monoclonal antibody (NPM, 1 : 1000) overnight, followed by incubation with a fluorescence-conjugated secondary antibody for 1 h, and finally dehydrated and mounted. The fluorescent signal was observed under fluorescent microscopy (Yun *et al*, 2003). For immunolabelling, lysates from the tissue samples were prepared as reported previously (Yun *et al*, 2003; Miao *et al*, 2006). One hundred micrograms of each lysate was separated by SDS–PAGE. The proteins were transferred onto blotting membranes. After blocking, the membranes were incubated overnight with mouse monoclonal antibody against NPM, rabbit polyclonal antibody against PCNA (FL-261, Santa Cruz Inc., Santa Cruz, CA, USA), and mouse monoclonal antibody against GAPDH (Kangchen Biotech, Shanghai, China) (NPM, 1 : 2000; PCNA, 1 : 1000; GAPDH, 1 : 1000), followed by incubation with a horseradish peroxidase-conjugated IgG. The blots were then visualised with an ECL kit (Amersham Life Science, Piscataway, NH, USA) and exposed for 30 s (NPM) and 1 min (PCNA, GAPDH) to X-ray film. The bands were analysed using the Quantity One™ Software (Bio-Rad, Hercules, CA, USA).

### Reverse transcriptase–PCR

Total RNA was extracted from 10 paired samples of frozen HCC tissue and adjacent hepatic tissue using the Trizol method (Gibco, Carlsbad, CA, USA) according to the manufacturer's instructions. One microgram of RNA sample was reverse transcribed with oligo(dT)15 primers (Promega, Madison, WI, USA) to obtain single-stranded cDNA. One-tenth of the product was used as template in PCR amplification for 28 cycles in a thermal cycler. Each cycle consisted of 30 s denaturation at 94.5°C, 30 s annealing at 55°C, and 1 min extension at 72°C. Under these conditions, the amplifications occurred in a linear exponential phase. The following primers were used: NPM forward, 5′-CAC CCG ATG GAA GAT TC-3′; NPM reverse, 5′-GGA CAG CCA GAT ATC AAC T-3′; G3PDH forward, 5′-AAA TCC CAT CAC CAT CTT CC-3′; and G3PDH reverse, 5′-TCC ACC ACC CTG TTG CTG TA-3′. The PCR products were analysed by 1.0% agarose gel electrophoresis. The abundance of PCR signals was determined using the Quantity One™ Software (Bio-Rad, Hercules, CA, USA).

### Immunohistochemistry

One hundred thirty-two sample blocks were sectioned for immunohistochemistry of NPM and PCNA. The above tissue array sections were prepared for immunohistochemistry of NPM with a three-step immunoperoxidase method using a Strept-Avidin Biotin kit (Dakopatts, Glostrup, Denmark) as previously described (Yun *et al*, 2003, 2004). Briefly, after blocking, the sections were incubated in primary antibodies overnight (NPM, 1 : 400; PCNA, 1 : 400), followed by incubation in secondary antibodies, and further incubation with the Strept-Avidin Biotin complex (Dakopatts, Denmark). Reactivity was developed in chromogen DAB solution. After contrast staining, the sections were dehydrated and mounted. A brown particle in a cell was considered as positive labelling. These sections were observed under light microscopy and the staining intensities were assessed by two pathologists – Dr JP Yun and Dr J Xiang.

### Statistical analysis

After immunohistochemistry of NPM and PCNA of tissue from cases with liver diseases, four types of nuclear staining intensities were discovered. These were classified as strongly positive (+++), intermediately positive (++), weakly positive (+), or negative (−). For each case, 1000 cells in one case were randomly selected and counted, scoring for these four intensities. For factor analysis, the percentages of the four intensities were transformed to one Ridit value for each case that was representative of the NPM or PCNA expression intensity. The usual tests (comparison of means, *t*-test, and one-way ANOVA) were then used to analyse the differences of NPM or PCNA expression among the different groups. The Pearson correlation was employed to examine the relationship between NPM and PCNA expression. Statistical significance was initially set at *P*<0.05.

## RESULTS

### Nucleophosmin expression in the HCC tissues

**Figure 1:**
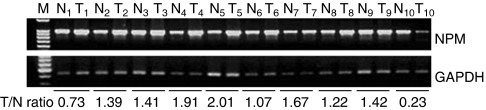
Nucleophosmin expression was detected in clinical samples with HCC by RT–PCR. Ten paired samples of HCC tissue and the corresponding liver tissue adjacent to the tumour were used. Samples T1–10 are HCC tissues, whereas N1–10 designate the corresponding adjacent healthy tissue samples. M: marker. Expression of the housekeeping gene, GAPDH, serves as a control.

In seven out of 10 cases, NPM mRNA expressions were higher in HCC tissues than in the paired liver tissue adjacent to the tumours (samples 2–5 and 7–9), as determined by RT–PCR (Figure 1). In three cases, NPM expressions of the HCC tissues were found to be lower than those in the adjacent healthy tissues (samples 1 and 10) and nearly same as that in the adjacent healthy tissue (sample 6). The expression levels of NPM in these samples with HCC were on average 1.31-fold higher than in the paired healthy tissue. These results indicated that NPM mRNA was expressed at a higher level in HCC than in the hepatocytes adjacent to the tumour.

**Figure 2:**
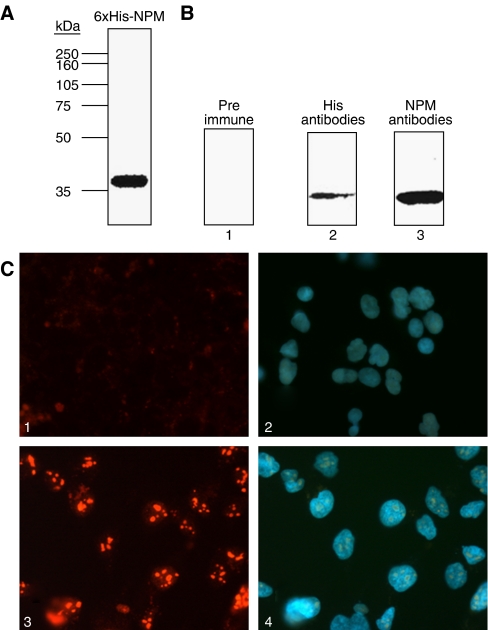
Characterisation of NPM antibodies. (**A**) The identity of NPM recombinant protein (1 *μ*g) was verified by Western blot analysis with an anti-NPM antibody. (**B**) The immunoreactivity of NPM protein to different antibodies (10 ng per lane) was analysed by Western blot using preimmune serum (lane 1), an anti-His antibody (1 : 4000, lane 2), or anti-NPM antibody (1 : 2000, lane 3). The recombinant NPM proteins were detected by His antibody or NPM antibody, but not by the preimmune serum. (**C**) Immunocytofluorescence analysis using NPM antibodies. Image 1: cultured Hep B3 cells were stained with preimmune serum; image 2: the same cells as in image 1 were stained with DAPI; image 3: cultured Hep B3 cells were stained with anti-NPM antibodies; image 4: the same cells as in image 3 were stained with DAPI. (All fields × 400.)

For use in immunolabelling studies, NPM monoclonal antibodies were raised against recombinant NPM protein (with a molecular weight of approximately 35 kDa) and characterised by Western blot and immunocytofluorescence (Figure 2). In total, one clone was screened to produce specific monoclonal antibodies against NPM that were used to immunolabel this protein in Western blot analysis, immunofluorescence, and immunohistochemistry.

**Figure 3:**
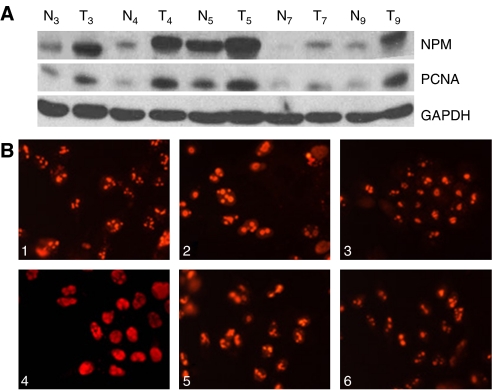
Nucleophosmin protein expression was detected in clinical samples with HCC by Western blot analysis and immunocytofluorescence. (**A**) Higher expression of NPM was found in HCC tissue samples (T3–5, T7, and T9) than in the corresponding adjacent tissue (N3–5, N7, and N9). This was similar to the PCNA expression trend in the same samples. As a control, GAPDH was used. (**B**) Nucleophosmin subcellular localisation was also examined in these cells. Hep 3B (image 1), PLC/PRF/5 (image 2), Huh-7 (image 3), Hep G2 (image 4), SK-Hep-1 (image 5), and Chang (image 6). (All fields, × 400.)

Nucleophosmin protein expressions in the above 10 paired samples were analysed by Western blot analysis. In seven out of 10 samples, expression of this protein was apparently higher in HCC tissues than in the liver tissue adjacent to the tumour; however, there was no apparent difference of expression in three cases. The mean expression level of the HCC samples was 1.88-fold higher than the paired liver tissue. The expression pattern of NPM was similar to that of PCNA (Figure 3A). These results indicate that NPM protein is expressed at a higher level in HCC than in hepatocytes adjacent to the tumour.

The subcellular expression of NPM was determined by immunocytofluorescence. It was found to localise predominantly in the nucleoli of six cultured cell lines and weakly in the nuclei of some cells such as Hep G2, PLC/PRF/5, SK-Hep-1, and Hep-3B (Figure 3B). This may suggest that NPM functions primarily in the nuclei of these cultured hepatoma.

### Expression of NPM and/or PCNA in HCC and non-malignant liver diseases

**Figure 4:**
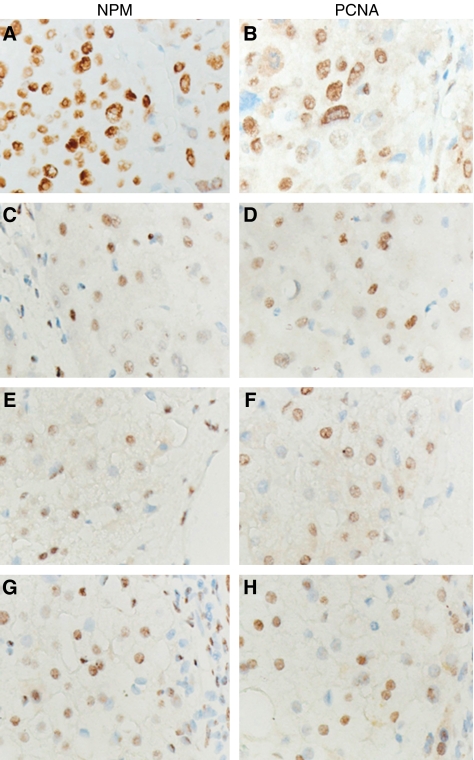
Nucleophosmin expression was observed in HCC and non-malignant diseases by immunohistochemistry and compared to PCNA expression. Nuclear expression of both NPM and PCNA was stronger in the HCC tumour cells than in hepatocytes with non-malignant diseases. Images A and B: immunoreactivity of NPM (**A**) and PCNA (**B**) in the tumour cells with HCC; images C and D: immunoreactivity of NPM (**C**) and PCNA (**D**) in the hepatocytes adjacent to the tumours with HCC; images E and F: Immunoreactivity of NPM (**E**) and PCNA (**F**) in hepatocytes with nodular hepatic hyperplasia; images G and H: immunoreactivity of NPM (**G**) and PCNA (**H**) in hepatocytes adjacent to tumours with haemangioma. All sections were counterstained with haematoxylin. (All fields × 400.)

**Figure 5:**
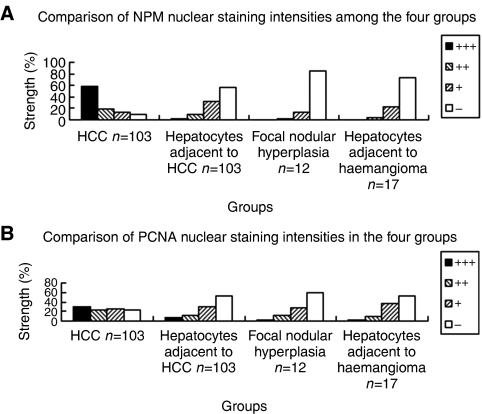
The staining intensities of NPM and PCNA were measured and calculated among the four groups including HCC and non-malignant liver diseases. (**A**) The median staining intensity percentages of NPM nuclear expression in the four groups are shown. (**B**) The median percentages of PCNA staining intensity in the four groups are shown.

As shown in Figures 4 and 5, NPM was more highly expressed in HCC (group 1), as determined by immunohistochemistry, was mainly located in the nuclei of the cancer cells, and was higher than that in the hepatocytes with non-malignant diseases (groups 2–4) in staining intensity or the total positive cell number. Counting the positive cells, scored for the four staining intensities, the median percentages of NPM expression in these groups are listed in Figure 5A.

The PCNA expression levels in the four groups were also determined and analysed by immunohistochemistry (Figures 4 and 5). It was found that PCNA labelling in the cancer cells with HCC was stronger than in the hepatocytes with non-malignant diseases. The median percentages of PCNA expression divided into the four groups are listed in Figure 5B.

To statistically compare NPM or PCNA expression among the four groups, staining intensity group percentages were transformed to Ridit values representative of NPM or PCNA expression in each case. The mean Ridit values of NPM or PCNA expressions in the four groups are listed in Table 1. There were significant statistical differences of NPM and PCNA expressions among the four groups by ANOVA analysis (*P*=0.000). Using the Student –Newman–Keuls analysis, NPM or PCNA expressions in HCC were significantly higher than in non-malignant diseases (*P*<0.05).

**Figure 6:**
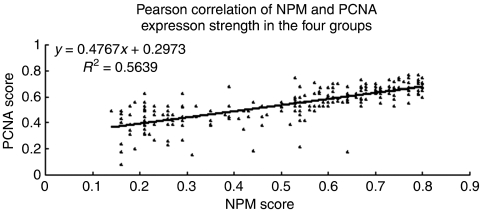
Potential correlations with NPM and PCNA expression were statistically analysed among the four groups including HCC and non-malignant liver diseases. Pearson correlation of NPM and PCNA expression revealed a correlation of expression among the four groups (*R*^2^=0.5639).

We also calculated the correlation of the mean Ridit values of NPM and PCNA expression by Pearson correlation analysis (Figure 6). There was statistical correlation between NPM and PCNA expression in the four groups (*R*^2^=0.5639).

### Statistic analysis on NPM expression differences in HCC with clinical parameters

We further investigated the differences of NPM or PCNA expression in 103 cases of HCC based on different clinical parameters, including the patient's sex, age, clinical TNM stage, tumour size, tumour amount, serum level of alpha fetal protein (AFP), histological grade, and the presence or absence of liver cirrhosis. These cases were divided into two groups for each clinical parameter (Marrero *et al*, 2005). The mean Ridit values of each group for each clinical parameter were measured by Ridit analysis and are shown in Table 2. The differences in the Ridit values for the two groups of each clinical parameter were analysed by *t*-test analysis. Significant statistical differences were found for three of the clinical parameters, including serum level of AFP, histological grade, and presence of liver cirrhosis (*P*<0.05). These data indicate that the levels of NPM expression in HCC correlate with the grading of the tumour, and thus upregulation of NPM may be involved in tumour progression. There were no statistical differences with respect to NPM expression in the HCC cases with the other clinical parameters including patient's sex, age, clinical TNM stage, tumour size, and tumour amount (*P*>0.05).

The differences of PCNA expression in the HCC cases based on the same clinical parameters were analysed and are shown in Table 2. We found no statistical differences in PCNA expression in the HCC cases with these clinical parameters (*P*>0.05).

### Nucleophosmin expression in human normal adult and embryo tissues

**Figure 7:**
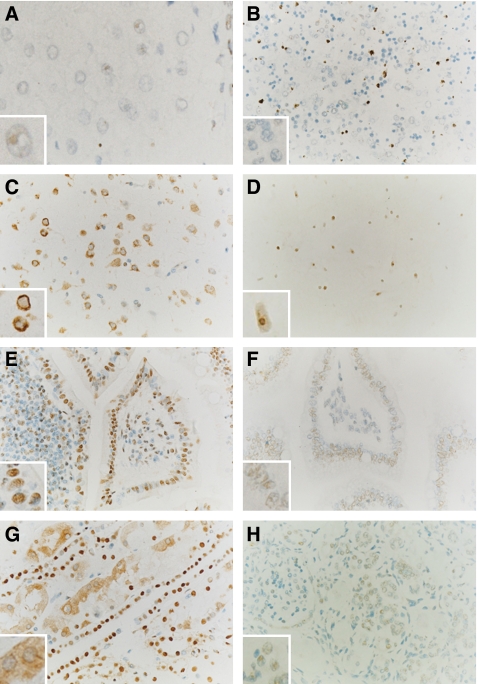
Nucleophosmin expression was detected in human adult and embryo tissues from different organs by immunohistochemistry. (**A**) Adult liver, (**B**) embryo liver, (**C**) adult brain, (**D**) embryo brain, (**E**) adult small intestine, (**F**) embryo small intestine, (**G**) adult kidney, and (**H**) embryo kidney. We observed different subcellular localisation of NPM in human cells, such as in the nucleoli of hepatocytes in normal adult liver (**A**), the nuclei of epithelia and glands (**D**–**F** and **H**), and the cytoplasm of adult cerebral neuronal cells (**C**) and epithelial cells of the kidney proximal tubule (**G**). There was very weak labelling in embryo liver hepatocytes (**B**). A greater magnification of the stained cells selected from the corresponding image is shown as inset. All sections are counterstained with haematoxylin. (All fields, × 400; inset images, × 1000.)

To evaluate the potential role of NPM in normal human cells, we investigated NPM expression in normal human adult and embryo tissues (5 months) by tissue array and immunohistochemistry. As shown in Figure 7, NPM expressions in different tissues were not quite consistent either in staining intensity or in location. Nucleophosmin was expressed in some cells from adults that did not express this protein in embryos. There were three types of NPM subcellular labelling: one was predominantly located in nucleoli, such as in normal adult liver (Figure 7A); another was mainly in the nuclei, such as in the epidermis, epithelia, glands, and proliferating lymphocytes located in the germinal centres (Figure 7D–H); the third type showed mainly cytoplasmic staining, such as in mitotic and some non-mitotic cells, such as adult cerebral neuronal cells (Figure 7C) and the epithelial cells of the kidney's proximal tubule (Figure 7G), embryo osteoblasts, and thymic cells. Interestingly, there was only very weak labelling of NPM in the hepatocytes from normal embryo livers (Figure 7B) and expression was found in the nucleolus and nucleus of the embryo brain cells (Figure 7D). Differential expression of NPM in normal human cells demonstrated that it may correlate with cell proliferation and differentiation.

### Nucleophosmin expression in cancer tissue

**Figure 8:**
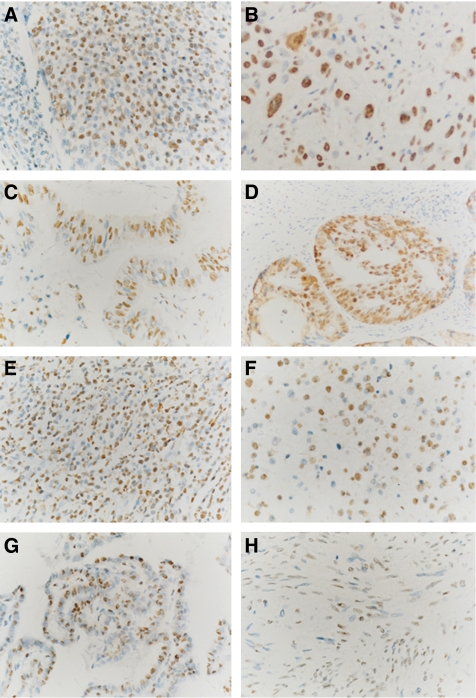
Nucleophosmin expression was observed in different types of human malignancies by immunohistochemistry. (**A**) Poorly differentiated squamous cervical carcinoma, (**B**) pulmonary squamous carcinoma, (**C**) adenocarcinoma of the small intestine, (**D**) adenocarcinoma of the prostate, (**E**) B-cell lymphoma of the testis, (**F**) astrocytoma of the brain, (**G**) thyroid papillary carcinoma, and (**H**) Wilms' tumour of the kidney. Strongly stained NPM nuclear expression was observed in the tumour cells of many malignancies (**A**–**G**), whereas only weakly stained nuclear expression was found in the Wilms' tumour sample (**H**). All sections are counterstained with haematoxylin. (All fields × 400.)

To evaluate the potential role of NPM in the human malignancies, we further investigated NPM expression in different cancer tissues by immunohistochemistry on tissue arrays. Strong nuclear expression of NPM was found in carcinomas originating from stratified epithelia derived from several organs, including the skin, oesophagus, cervix, and lung, in carcinomas from glandular epithelia from the breast, thyroid gland, lung, digestive tract, ovary, and prostate, and in other malignancies such as B-cell lymphoma, renal cell carcinoma, and glioblastoma. However, we observed only nucleolar expression and weak nuclear expression in Wilms' tumour of kidney. The NPM expressions in eight different malignancies originated from eight different organs are shown in Figure 8. In general, stronger nuclear staining of NPM expressions in the malignancies was observed as compared to those in the corresponding normal tissues (Figure 7; data not shown). Put together, NPM expression detected in human malignancies implicated a potentially important role for NPM in tumorigenesis and development.

## DISCUSSION

The present study revealed four principal findings: (1) that NPM is differentially expressed, either in quantity or subcellular location, in malignant human liver cancer (HCC) compared to hepatocytes with non-malignant liver diseases. Similar differences in PCNA expression were detected among these cases. These results indicate that NPM might play an important role in the genesis and development of HCC. We also found that (2) in the cases with HCC, NPM overexpression was correlated with clinical parameters, such as serum AFP level, tumour pathological grading, and cirrhosis, suggesting that NPM might serve as a potential marker for HCC. (3) Nucleophosmin was differently expressed either in quantity or in subcellular location in normal human liver and other tissues when adult and embryo tissues were compared. The NPM expression of normal human cells was shown to correlate with cell proliferation and differentiation. Lastly, (4) NPM was found to be highly expressed in multiple cancers from different organs derived from either epithelial or non-epithelial origins. This NPM expression in multiple human malignancies implicates that NPM might play an important role in tumorigenesis and development.

The present study examined the potential role of NPM in HCC. Our data revealed a high level of NPM expression in the majority of HCC examined. Overexpression of NPM in HCC may thus represent an acquired phenotypic feature. Furthermore, there was a positive correlation between NPM expression and tumour pathological grading or HCC with liver cirrhosis. Therefore, NPM may be an important factor in the development of HCC. Previous studies showed that NPM seemed to be associated with both oncogenic activity and tumour suppression. Hence, overexpression of NPM in NIH3T3 cells resulted in malignant transformation (Kondo *et al*, 1997). Nucleophosmin nuclear overexpression in colon adenoma/carcinoma seems also to be associated with adenoma/carcinoma progression (Nozawa *et al*, 1996). It has been shown that NPM overexpression was present in several human solid tumours, including gastric cancer (Tanaka *et al*, 1992), colon cancer (Nozawa *et al*, 1996), bladder cancer (Tsui *et al*, 2004), prostate cancer (Subong *et al*, 1999), thyroid cancer (Onda *et al*, 2004), and ovary cancer (Zhang, 2004). Interestingly, a recent review reported that NPM overexpression in tumour cells led to increased proliferation and inhibition of apoptosis and was involved in human tumorigenesis (Grisendi *et al*, 2006).

The present study also demonstrated that there were similarities of NPM nuclear expression and PCNA expression between HCC and non-malignant diseases. We recently observed that NPM expression in rat hepatocytes increased quantitatively and changed in subcellular location during liver regeneration after partial hepatectomy. Moreover, these alterations of hepatocyte NPM level were consistent with different phases of the cell cycle and the level of PCNA (Yun *et al*, 2002, 2003). Yung's group has recently shown that the expression of NPM correlates with PCNA expression. They demonstrated that the increased NPM resulted in enhanced DNA repair ability and rescued cells from UV killing (Wu *et al*, 2002). They further provided evidence that ectopic overexpression of NPM caused a marked upregulation in the amounts of PCNA and YY1 (a multifunctional transcriptional factor) proteins. Nucleophosmin regulates the PCNA promoter through YY1 (Weng and Yung, 2005).

Another interesting finding in this study is that there are different subcellular locations of NPM in the normal cells of the embryo and the adult. We describe three types of NPM cellular distribution found in normal human adult or embryo tissues – the nucleoli, nuclei, or the cytoplasm. Differential subcellular expression of NPM in normal human cells may correlate with cell proliferation and differentiation. Previous reports have shown that NPM is involved in the assembly and/or intranuclear transport of pre-ribosomal particles and in cytoplasmic/nuclear trafficking (Spector *et al*, 1984; Yung *et al*, 1985; Borer *et al*, 1989). In mitotic cells, most nuclear proteins were dispersed throughout the cytoplasm, including NPM, which then re-localised to the centrosomes, and later to each daughter cell that received one centrosome bound by NPM after mitosis (Okuda *et al*, 2000). Interestingly, we also found that some of the non-mitotic cells, such as adult cerebral neuronal cells, epithelial cells of the adult kidney proximal tubule, and embryo osteoblast and thymic cells contain cytoplasmic NPM. The functional significance and the mechanism behind this cytoplasmic localisation remain to be determined. Previous studies found that two isoforms of NPM, termed B23.1 and B23.2, were expressed in different subcellular locations of human cells and possessed different functions (Chang and Olson, 1990; Umekawa *et al*, 2001). B23.1 was primarily localised in nucleoli, where it was likely targeted to rRNA-rich nucleoli, possibly owing to its RNA-binding activity, whereas B23.2 was localised in the nucleoli and also throughout the nucleoplasm; the function of B23.2 is not yet well understood. These two proteins are identical except for the C-terminal 35 amino acids observed in B23.1 that is absent in B23.2. The expression level of B23.2 was much lower than that of B23.1. Interestingly, the expression level of B23.1 was closely correlated with the cell growth rate, whereas that of B23.2 remained relatively constant (Wang *et al*, 1993).

Another previous study demonstrated the cytoplasmic location of NPM and the mutation of exon 12 of the NPM1 gene as a hallmark of a large subgroup of primary acute myelogenous leukaemia (NPMc+ AML) (Falini *et al*, 2005). However, we have not observed cytoplasmic NPM in any of the 103 cases of HCC or other cancers in the tissue array used in the present study. The cytoplasmic location in the solid cancers will be worthy of further study.

Both polyclonal and monoclonal antibodies to NPM have been reported in the past, but only a few immunocytochemical analyses or distribution studies of NPM in human tissues have confirmed its ubiquitous expression in both normal and neoplastic cells (Nozawa *et al*, 1996; Cordell *et al*, 1999; Subong *et al*, 1999). The study describes monoclonal antibodies that detect NPM in paraffin-embedded tissue array samples and confirms NPM distribution within different cells.

Altogether, the present study provides evidence that NPM may be involved in hepatocarcinogenesis and serve as a potential marker for applied clinicopathology in HCC.

## Figures and Tables

**Table 1:** Comparison of NPM and PCNA expressions among the four groups

		**NPM expression**			**PCNA expression**		
			**ANOVA**			**ANOVA**	
	**Cases**	**Ridit (mean±s.d.)**	**F**-**value**	***P*-value**	**Ridit (mean±s.d.)**	**F-value**	***P*-value**
Group 1	103	0.295±0.115			0.429±0.107		
Group 2	103	0.679±0.087			0.624±0.084		
Group 3	12	0.603±0.097			0.608±0.085		
Group 4	17	0.708±0.065	285.14	0.000^*^	0.650±0.048	87.184	0.000^*^
Total	235	0.509±0.214			0.540±0.135		

NPM=nucleophosmin; PCNA=proliferative cell nuclear antigen; ANOVA=analysis of variance.

Group 1: HCC; group 2: hepatocytes adjacent to the tumours with HCC; group 3: hepatocytes in the lesions with focal nodular hyperplasia; group 4: hepatocytes adjacent to the tumours with haemangioma. ^*^Statistically significant (*P*<0.01).

**Table 2:** NPM and/or PCNA expressions in HCC with clinical parameters

		**NPM expression (mean±s.d.)**			**PCNA expression (mean±s.d.)**		
	**Cases (*n*)**	**Ridit value**	***T*-value**	***P*-value**	**Ridit value**	***T*-value**	***P*-value**
*Sex*	103						
Male	96	0.294±0.107			0.431±0.104		
Female	7	0.229±0.141	1.53	0.129	0.390±0.150	0.713	0.501
							
*Age*	103						
<50	61	0.291±0.110			0.418±0.100		
⩾50	42	0.288±0.112	0.165	0.87	0.444±0.117	1.202	0.508
							
*TNM stage* a	89						
I–II	30	0.312±0.102			0.443±0.110		
III–IV	59	0.275±0.111	1.542	0.127	0.428±0.100	0.677	0.5
							
*Tumour size*	103						
<5 cm	35	0.309±0.108			0.430±0.114		
⩾5 cm	68	0.2800±0.111	1.248	0.215	0.428±0.105	0.105	0.707
							
*Tumour amount*	103						
1	80	0.300±0.110			0.432±0.103		
⩾2	23	0.254±0.108	1.787	0.077	0.418±0.125	0.516	0.607
							
*Serum AFP*	89						
<20 ng ml^−1^	27	0.323±0.107			0.445±0.085		
⩾20 ng ml^−1^	62	0.272±0.107	2.088	0.04^*^	0.428±0.110	0.734	0.366
							
*HBsAg*	89						
Positive	82	0.284±0.109			0.431±0.106		
Negative	7	0.326±0.108	0.982	0.329	0.451±0.065	0.491	0.313
							
*Histological grade* b	103						
I	16	0.349±0.092			0.461±0.088		
II–IV	87	0.279±0.111	2.376	0.019^*^	0.423±0.110	1.325	0.161
							
*Liver cirrhosis*	103						
Yes	73	0.274±0.103			0.430±0.111		
No	30	0.328±0.120	2.145	0.037^*^	0.426±0.101	0.152	0.88

NPM=nucleophosmin; PCNA=proliferative cell nuclear antigen; HCC=hepatocellular carcinoma; AFP=alpha fetal protein.

a TNM stage was based on the UICC criteria published in 1997.

b Histological grade was with reference to WHO classification published in 2000.

* Statistically significant (*P*<0.05).
